# The role of the sensory input intervention in recovery of the motor function in hypoxic ischemic encephalopathy rat model

**DOI:** 10.1152/jn.00054.2024

**Published:** 2024-04-03

**Authors:** Juchuan Dong, Yifei Dong, Lijuan An, Yufan Wang, Yongmei Li, Lihua Jin

**Affiliations:** ^1^Department of Rehabilitation Medicine, Second Affiliated Hospital of Kunming Medical University, Kunming, People’s Republic of China; ^2^Department of Rehabilitation Medicine, Kunming Medical University, Kunming, People’s Republic of China

**Keywords:** enriched environment, hypoxic ischemic encephalopathy, motor function, neurologic function, sensory input

## Abstract

Motor disturbances predominantly characterize hypoxic-ischemic encephalopathy (HIE). Among its intervention methods, environmental enrichment (EE) is strictly considered a form of sensory intervention. However, limited research uses EE as a single sensory input intervention to validate outcomes postintervention. A Sprague–Dawley rat model subjected to left common carotid artery ligation and exposure to oxygen-hypoxic conditions is used in this study. EE was achieved by enhancing the recreational and stress-relief items within the cage, increasing the duration of sunlight, colorful items exposure, and introducing background music. JZL184 (JZL) was administered as neuroprotective drugs. EE was performed 21 days postoperatively and the rats were randomly assigned to the standard environment and EE groups, the two groups were redivided into control, JZL, and vehicle injection subgroups. The Western blotting and behavior test indicated that EE and JZL injections were efficacious in promoting cognitive function in rats following HIE. In addition, the motor function performance in the EE-alone intervention group and the JZL-alone group after HIE was significantly improved compared with the control group. The combined EE and JZL intervention group exhibited even more pronounced improvements in these performances. EE may enhance motor function through sensory input different from the direct neuroprotective effect of pharmacological treatment.

**NEW & NOTEWORTHY** Rarely does literature assess motor function, even though it is common after hypoxia ischemic encephalopathy (HIE). Previously used environmental enrichment (EE) components have not been solely used as sensory inputs. Physical factors were minimized in our study to observe the effects of purely sensory inputs.

## Graphical abstract

**Figure:**
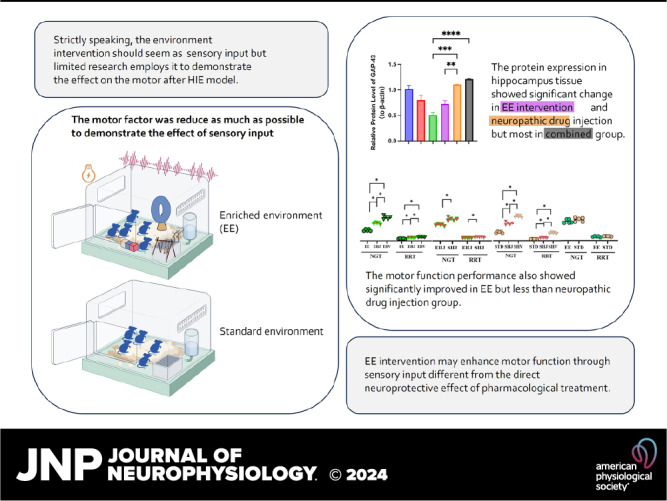

## INTRODUCTION

Hypoxia ischemic encephalopathy (HIE) occurs when the brain is exposed to hypoxia and ischemia, and can happen before birth, during labor and delivery, or after birth (1). Early treatment for brain injury after HIE, which lasts for weeks to years after the first incidence, appears to aid recovery (2). Identifying alternative early and effective neuroprotective treatments for HIE could substantially reduce the global disease burden due to HIE, and as such is of significant global interest.

Current treatments for HIE are unsatisfactory. Despite therapeutic hypothermia being the sole established remedy for moderate or severe HIE, patients still encounter significant complications (3, 4). Behavioral changes related to HIE are directly linked to decreases in brain volume and the shrinkage of certain brain structures. These changes are connected to cytotoxicity, oxidative stress, and apoptosis (5). Early intervention is crucial in HIE management, as it decreases complications and secondary injuries. However, most experiments focused on a single method and do not reflect the reality of a clinical situation. Moreover, although the HIE could lead to impairment of both movement and memory-related function, most research focused on the cognitive and memory-related outcomes rather than motor function especially the intervention of enriched environment (EE).

An EE comprising complex factors, such as physical, cognitive, and social stimuli, can improve rodent welfare (6). EE is commonly given immediately after injury or in the later phase (7). By enhancing neuroplasticity motor activity, sensory stimulation, and cognitive recovery, the utilization of these EE settings brings about a multitude of advantages (8, 9). EE paradigms include, but are not limited to, introducing cage mates and providing larger cages, various bedding types, platforms, and visual and tactile stimuli in the cages (9). Based on these explanations, it can be inferred that EE predominantly encompasses the sensory input aspect. Nevertheless, the majority of interventions in EE following HIE primarily assess cognitive-related facets, such as memory and executive function, rather than motor function.

Numerous studies have posited that the precise mechanisms elucidating the phenomenon of environmental enrichment (EE) remain incompletely comprehended (7, 10–12). Nonetheless, in the course of implementing interventions, factors beyond singular sensory inputs, particularly those associated with physical activity, have been integrated into EE interventions (7). Despite the deductions made by these investigations regarding the potential neuroprotective benefits and enhancement of synaptic plasticity associated with EE, these findings do not exclude the possibility that increased physical activity may also contribute to the effects on neural plasticity. If EE alone could produce these advantageous effects for HIE solely through sensory inputs, it could be deemed as an additional avenue for intervention in clinical practice.

We aimed to evaluate whether the intervention of sensory input could improve the motor function as much as the improvement of neuroprotection drug in HIE rat model.

## MATERIALS AND METHODS

### Animals

The experimental procedures were approved by the Institutional Animal Care and Use Committee of the Preclinical Research Institute of the Second Hospital of Kunming Medical University (KMMU20230233). Sprague–Dawley rats were purchased from Kunming Medical University Laboratory Animal Technology (Kunming, PR China). All rats were maintained on alternating cycles of 12-h light/dark with food and water available ad libitum. Temperatures alternated between 23°C and 25°C. A total of 30 rats were divided into EE and standard environment (STD) groups 7 days after postnatal day (PND) and were further divided into sham, HIE + vehicle, and HIE + JZL 184 [JZL, a selective, irreversible monoacylglycerol lipase (MAGL) inhibitor (13)] groups. All the rats in this study were male. Except for the sham operation group, the other groups underwent the hypoxia and ischemia protocols. Rats in the sham-operated group underwent wound ligation after isolation of the left common carotid artery (CCA).

### Hypoxia and Ischemia Protocols

Before inducing ischemia, rats were anesthetized using sevoflurane (5%) and placed supine on the operating table; to sustain the anesthesia, 2% sevoflurane was used (14). A core body temperature of 37°C was maintained using an automatic heating blanket during surgery. After disinfection with iodophor, a 2-cm port incision was introduced on the left side of the trachea using ophthalmic scissors. The muscle and glands were stripped using microsurgery forceps to expose the left CCA and were carefully separated from the adjacent vagus nerve. The telecentric and proximal ends of the CCA were ligated using 3-0 sutures (Johnson & Johnson), and the vessel was cut between the two points. Two to three drops of gentamicin (80,000 U) were added, and the wound was sutured. After the rats awakened from anesthesia, they returned to their mothers and were fed for 90 min. Subsequently, the rats were transferred into a hypoxic plexiglass chamber (8% O_2_, 92% mixed concentration gas, temperature of 36°C) for 1 h, following which the rats were transferred to their mothers for continued feeding.

### Drug Intervention Vehicle

JZL was selected in this study as a neuroprotection drug, and it is a selective inhibitor of MAGL, the enzyme responsible for 2-AG hydrolysis (15). The endocannabinoid 2-arachidonoylglycerol (2-AG) is a GABAergic messenger that controls synaptic transmission and plasticity (16). MAGL inhibition after JZL184 administration was rapid (maximal inhibition achieved within 0.5 h posttreatment) and potent (>80% inhibition of 2-AG hydrolysis activity, resulting in a 7- to 9-fold increase in brain 2-AG levels). The half-life of JZL is approximately 7 h (17). Both JZL subgroups in the EE and SE groups received equal volumes of vehicle of solubilizing (2 mg/kg body wt). Vehicle injections were performed 1 h before hypoxia and 24 and 48 h after the first injection.

### Intervention

**Figure 1.**
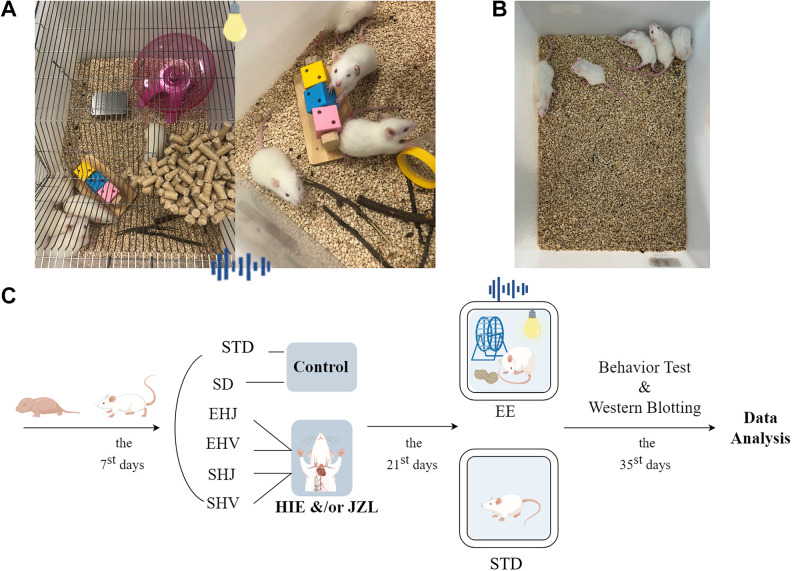
Experimental scheme. *A*: representative picture of an enriched environment (EE) cage. *B*: representative picture of a standard environment (STD) cage. *C*: timeline for the intervention experiment and evaluation. EHJ, enriched environment + hypoxic ischemic encephalopathy + JZL 184 (JZL) intervention; EHV, enriched environment + hypoxic ischemic encephalopathy + vehicle intervention; SHJ, standard environment + hypoxic ischemic encephalopathy + JZL intervention; SHV, standard environment + hypoxic ischemic encephalopathy + vehicle intervention. JZL, JZL intervention. Intervention timeline drawn by Figdraw. ID: TIYRO7d034.

Typically, rats exhibit ocular opening between 14 and 17 days postnatally. In light of the study’s focus on environment-related intervention such as visual stimuli, the intervention period for EE has been established as 21–35 days postnatally, encompassing a 14-day range. This timeframe also aligns with the 14–21 days post-HIE that was postacute status of period. The EE condition included visual stimulation such as colorful molar rods, swings, and running wheels. Furthermore, the experimental condition of the EE group involved subjecting the rats to a 6-h duration of white light exposure and continuous background music for auditory stimulation. To mitigate habituation, regular modifications are made to the colors, positions of the toys, and background music. Because the intervention method utilized in this study incorporates various environmental variables, significant efforts have been undertaken to mitigate potential biases stemming from housing conditions. Specifically, measures were taken to ensure that the two cohorts of rats were housed in distinct rooms, thereby minimizing the likelihood of cross-contamination between groups during exposure to auditory and visual stimuli. This approach served to reduce the risk of bias in our research. Behavioral tests for test cognitive and stress, motor functional tests, and Western blotting were performed once in both groups (Fig. 1).

### Behavior Tests

The standard hidden platform version of the Morris water maze (MWM) was used as previously described (18). The details are described in the Supplemental Materials. The movements of rats were tracked and analyzed using the VisuTrack system (Xin Run, Shanghai, PR China).

To assess stress-induced anhedonia, a sucrose preference test (SPT) was performed in a calm environment with dim illumination. The consumption and detail of data collection were performed as previously reported and are described as in the Supplemental Materials (19).

### Motor Function Test

The negative geotaxis test (NGT) was used to examine motor coordination in rats (20). The baby rats were positioned on an inclined surface, with their heads pointed downward. We recorded the length of time spent by the rats rotating their body such that they were facing upward (21).

The righting reflex test (RRT) is a simple and rapid test to assess motor neuron function such as locomotor abilities in rodent models (22). The rats were flipped onto their backs on a flat surface and the time required to right themselves was measured for at least two consecutive trials (23).

### Western Blotting

The rats were euthanized at 35 days after PND. Hippocampal tissue ipsilateral to the injury site was removed and homogenized in RIPA buffer supplemented with 1× protease inhibitor (Thermo Fisher Scientific, Rockford, IL). The protein concentration in each sample was quantified utilizing the bicinchoninic acid (BCA) method, and equal amounts of protein were loaded onto a 10% SDS-PAGE gel (Bio-Rad). Subsequently, the protein was transferred to the PVDF membrane, blocked with quick blocking solution (EpizymeBio, PR China) for 20 min at room temperature (21°C–25°C), and then incubated with the following primary antibodies overnight at 4°C: anti-rabbit growth-associated protein 43 (GAP43; 1:6,000; Cat. No. 16971-1-AP; Proteintech, PR China) and anti-mouse β-actin (1:50,000; Cat. No.: 66009-1-Ig; Proteintech, PR China). The level of GAP-43 may be related to GABAergic synapses (24), and Western blot analysis of GAP-43 protein expression was conducted to assess the impact of different interventions on the GABAergic synapse signaling pathway. After incubation with goat anti-rabbit or anti-mouse IgG (H + L)-HRP-conjugated secondary antibody (1:6,000; SA0000-1; Proteintech, PR China) for 2 h at room temperature, images were acquired using the ChemiDoc Touch imaging system (Bio-Rad) and densitometric analysis was performed using Fiji for Mac OS X.

### Statistical Analyses

Owing to the small sample size in each group, nonparametric tests were used. The statistical significance of the differences was evaluated using the Kruskal–Wallis test. Post hoc tests were conducted for multiple group comparisons using the Mann–Whitney test. Western blot data were analyzed for normality using the Shapiro–Wilk test for normality and one-way analysis of variance (ANOVA) for multigroup comparisons. All statistical analyses were performed using SPSS version 26 (IBM, Armonk, NY), and statistical significance was set at *P* < 0.05. Data were plotted using the GraphPad Prism 8.0 program (GraphPad Inc., San Diego, CA).

## RESULTS

### Behavior Test

**Figure 2.**
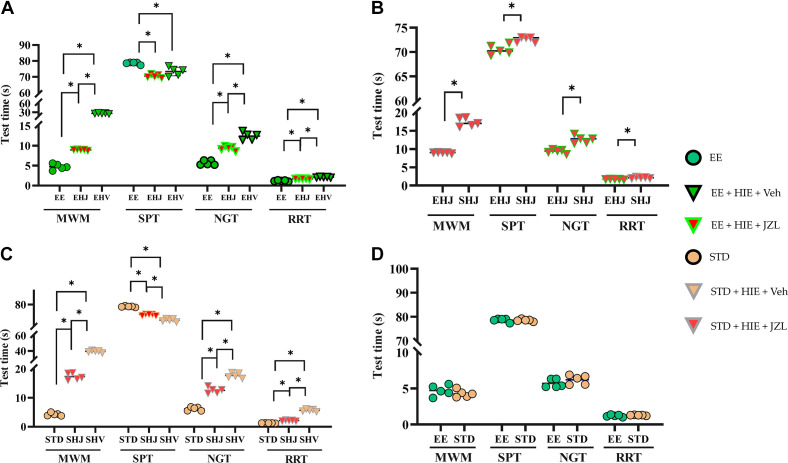
Effectiveness of different environments and interventions. Different interventions under the enriched environment (EE) condition (*A*); different environments after HIE and JZL interventions (*B*); different interventions under the standard environment (STD) condition (*C*); different environments in the control group (*D*). EHJ, enriched environment + hypoxic ischemic encephalopathy + JZL 184 (JZL) intervention; EHV, enriched environment + hypoxic ischemic encephalopathy + vehicle intervention; SHJ, standard environment + hypoxic ischemic encephalopathy + JZL intervention; SHV, standard environment + hypoxic ischemic encephalopathy + vehicle intervention. JZL, JZL intervention. *P < 0.05.

Both of the MWM time and SPT time in the only EE or only JZL were significantly different than control group, but MWM time in the EE combined with JZL injection group was significantly shorter than that in the other groups, except for the control group (*P* < 0.05; Fig. 2).

### Motor Function Test

Both NGT and RRT times were significantly shorter in the single EE intervention or single JZL injection group, especially in combination with EE, in which the rats exhibited a shorter rotation time to the upward head position compared with those in both the vehicle injection and standard environment (STD) condition groups (*P* < 0.05; Fig. 2).

### EE Did Not Prime under Normal Neurological Function

In the control group, the NGT, RRT, MWM, and SPT scores did not change between the EE and STD conditions (*P* > 0.05; Fig. 2*D*).

### Western Blotting Results

**Figure 3.**
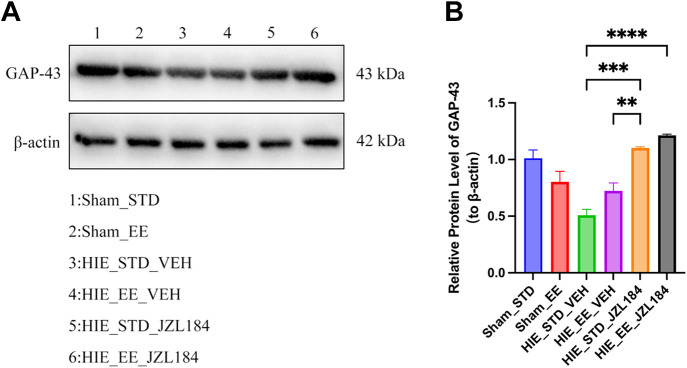
Quantified GAP-43 protein expression in hippocampus tissue. *A*: Western blots analysis showing the GAP-43 protein in each group after intervention. *B*: means of the staining intensity ratios of GAP-43 in each group. EE, enriched environment; STD, standard environment; EHJ, enriched environment + hypoxic ischemic encephalopathy + JZL 184 (JZL) intervention; EHV, enriched environment + hypoxic ischemic encephalopathy + vehicle intervention; SHJ, standard environment + hypoxic ischemic encephalopathy + JZL intervention; SHV, standard environment + hypoxic ischemic encephalopathy + vehicle intervention. JZL, JZL intervention. Values are expressed as means ± SD; **P < 0.01, ***P < 0.001, ****P < 0.0001.

The results showed a decrease in GAP-43 expression in the HIE_STD_VEH group compared with the sham group. Interestingly, treatment with either EE or JZL alone resulted in an increase in GAP-43 expression. Furthermore, a synergistic effect was observed when EE and JZL were combined, leading to a further enhancement of GAP-43 expression (Fig. 3, *A* and *B*).

## DISCUSSION

In this study, the independent application of EE and the sole use of JZL both ameliorated the cognitive deficits resulting from HIE damage. Moreover, alterations in the levels of GAP43 in the hippocampus corroborate that both sensory stimulation and neuroprotective pharmaceuticals can enhance cognitive functions. In addition, these two interventions also improved motor functions, and their combined use was found to be more efficacious.

The drug of neuroprotective measures could mitigate neuroinflammation and oxidative harm thereby safeguarding neurons to enhance cognitive function (25). Alternatively, it could also be attributed to the augmentation of synaptic plasticity, which subsequently enhances cognitive functions related to memory (26, 27). In this study, the JZL itself is a MAGL inhibitor, and MAGL degrades 2AG (15). The endocannabinoid 2AG is a GABAergic messenger that controls synaptic transmission and plasticity (16). Which means that, the improvement of motor functions in this study can be ascribed to neuroprotection that fortifies neural pathways, thereby enhancing motor abilities, moreover, it may be achieved through the promotion of synaptic plasticity (28).

The effect of EE intervention, what focus on the sensory input, the enhancement of cognitive functions is achieved through the reinforcement of neural network connections during the process of recognizing inputs such as colors and sounds, thereby promoting increased neuronal connectivity (29). In addition, sensory input has the potential to augment attentional control capabilities in rats, consequently leading to improved cognition (30). The amelioration of motor functions through sensory training may be attributed to the integration of multisensory information, resulting in enhanced motor control and the refinement of movement coordination and precision (31).

Both intervention methods have the potential to enhance cognition and motor functions through distinct pathways, making them promising for addressing deficits associated with cognitive and motor impairments following HIE. The concurrent utilization of both methods appears to activate multiple pathways simultaneously, leading to more significant improvements in cognitive and motor functions. However, it is unfortunate that our study did not investigate brain region connectivity, necessitating further research to substantiate these hypotheses.

Many studies have used movement devices, such as running wheels, in EE cages; however, this might increase bias as the additional intervention of physical exercise itself could increase cerebral integrity and neurocognition (7). We attempted to decrease the effects of additional devices and compared the two groups in a relatively similar space, but increased environmental factors such as music and light because exposure to flickering light could reduce amyloid plaques in an animal model with cognitive impairment (32). The results showed significant change of both cognitive and motor after sensory input intervention even the factors of motor intervention are reduced as low as achievable.

In previous study related to the effect of EE in HIE animal model, the EE protocol included larger cages than those for the control group (allowing for a greater range of activity) and more exercise equipment (providing more opportunities for physical intervention) (7, 11, 12). These interventions, even when implemented during crossover experiments, consistently demonstrated sustained functional improvements during standard environment in HIE rats. However, in other target populations such as poststroke models (33), the EE intervention did not demonstrate a statistically significant improvement when without neurological injury, as indicated by our study findings. This result could explain as, in rat models that did not have neural damage, the standards of the EE were higher than those for rats with damage. Additional methods, such as cerebral bioenergetics or other brain functions, may be required. So, these observed enhancements reported in previous studies may not be solely attributed to sensory input, but rather may be influenced by other factors contributing to the sustained improvement in functionality. The potential confounding effect resulting from heightened physical intervention cannot be disregarded. In our investigation, we endeavored to mitigate the impact of physical activity to exclusively examine the ramifications of sensory stimuli, such as sound, light, and color, on HIE rats. Such interventions may prove more appropriate for clinical rehabilitation approaches. Nevertheless, as we did not establish the interconnections among brain regions, our findings can merely offer guidance for clinical practice, suggesting the potential for stimulating multiple pathways through both of drug and environmental intervention modalities to hasten symptom amelioration following HIE.

This study is noteworthy due to previous research indicating that some of the combination of drug injection and environmental interventions may exhibit enhanced efficacy, although not all studies have demonstrated a synergistic effect (34, 35). Our findings suggest that our research approach has introduced a novel concept, specifically that JZL and real environmental factors may have an additive impact on the healing process. Subsequent studies may further explore additional proteins associated with synaptic pre- and postsynaptic neurotransmission, such as SYN (synaptic vesicle protein 2) or PSD-95 (postsynaptic density protein 95), in conjunction with neurofunctional data to elucidate the efficacy of some integrated therapeutic strategy. Given the lack of effective treatment options for HIE, these studies have the potential to be completed quickly and will pave the way for clinical trials in the coming years.

### Conclusions

Collectively, our findings suggest that the single sensory input intervention could promote the recovery of cognition and motor function, whereas combining with pharmacological treatment could yield superior outcomes. Further studies must be conducted to screen for environmental factors that favor neuroplasticity, as environmental interventions are more accessible than other invasive methods.

## DATA AVAILABILITY

The datasets used and/or analyzed for the development of this manuscript are available from the corresponding author on reasonable request.

## SUPPLEMENTAL DATA

10.6084/m9.figshare.25392622.v1 Supplemental Materials: https://doi.org/10.6084/m9.figshare.25392622.v1.

## GRANTS

This research was funded by the Yunnan Rehabilitation Clinical Medical Center Grant zx2019-04-02, Kunming Medical Graduate Education Innovation Fund 2023S319, and Yunnan Provincial Department of Education Grant 2024J0350.

## DISCLOSURES

No conflicts of interest, financial or otherwise, are declared by the authors.

## AUTHOR CONTRIBUTIONS

Y.D., Y.L., and L.J. conceived and designed research; J.D., L.A., and Y.W. performed experiments; Y.D. and L.J. analyzed data; Y.L. and L.J. interpreted results of experiments; J.D., Y.D., L.A., Y.L., and L.J. edited and revised manuscript; Y.L. and L.J. approved final version of manuscript.
